# Psychological relation of tandem nursing to children’s socio-emotional development and attachment to their caregiver

**DOI:** 10.1038/s41598-026-52462-2

**Published:** 2026-05-19

**Authors:** Jana Lund, Julian Trostmann, Louisa Kulke

**Affiliations:** 1 Department of Developmental Psychology with Educational Psychology, University of Bremen, Hochschulring 18, 28359 Bremen, Germany; 2 Kassel, Germany

**Keywords:** Tandem breastfeeding, Breastfeeding during pregnancy, Breastfeeding, Mother–child relationship, Sibling relationship, Parenting styles, Health care, Medical research, Psychology, Psychology

## Abstract

In tandem breastfeeding, a mother breastfeeds two children of different ages at the same time. Although it occurs regularly, there is little research on tandem-breastfeeding and its psychological aspects. The present set of two studies fills this research gap. Study 1 follows a qualitative approach to generate relevant psychological topics related to tandem breastfeeding through semi-structured interviews with tandem breastfeeding mothers and mothers who breastfed two children but not in tandem. In the quantitative Study 2, an online questionnaire was generated based on the relevant factors for tandem-breastfeeding identified in Study 1. We found that older children who were not breastfed in tandem changed their focus from the mother to the partner after birth of the younger sibling and rejected their mother, which was not the case for children who were breastfed in tandem. Tandem-breastfeeding mothers showed more attachment parenting and less authoritarian parenting. However, they felt increased anger and aggression towards the older child. The current set of studies provides the first scientific basis for research in psychological aspects of tandem nursing. It stresses the relevance of research in tandem breastfeeding to avoid adverse effects such as anger towards children, while promoting positive effects on the mother-child relationship.

**Supplementary Information:**

The online version contains supplementary material available at 10.1038/s41598-026-52462-2.

## Introduction

According to the current recommendation of the World Health Organization (WHO), infants should be breastfed exclusively up to the age of six months and supplementary up to the end of the second year of life and beyond^1^. If a child is breastfed by the mother for two years or longer, it is possible that the mother will become pregnant again during this time and give birth to a second child. If the mother wants to abide by the WHO recommendations, she would then continue breastfeeding her older child and thus breastfeed both siblings in parallel. Simultaneously breastfeeding two children of different ages is called tandem breastfeeding^2^. However, there is very little research on tandem breastfeeding and most studies on tandem breastfeeding follow a qualitative design with few participants.

In many European countries, such as Denmark, Sweden and Austria, the rate of children still being breastfed at six months increased in the 1980s and 1990s^3^. In Germany, the number of children who were fully breastfed up to the age of 12 months and for longer than 12 months is significantly higher in the 2013 and 2014 birth cohorts than among the children born in 2009 and 2010^4^. The data from these studies suggest that the duration of breastfeeding has continued to increase in recent decades and is closer to the current WHO recommendation on breastfeeding^1^. At the same time, the age gap between siblings in Germany shortened slightly from 2000 to 2010 (Geburtenfolge und Geburtenabstand, n.d.). These developments increase the likelihood that today a mother who is still breastfeeding her older child is already pregnant with a younger child and wants to continue breastfeeding her older child. Tandem breastfeeding has so far been little researched^5^ and therefore little can be said about possible psychological effects of tandem breastfeeding on the mother and the children.

According to the WHO, breast milk is recommended for feeding infants^1^. Breast milk contains antibodies that protect the child from several common childhood diseases and children who are breastfed are less likely to suffer from obesity or overweight and also from diabetes later in life^1^. Breastfeeding and the duration of breastfeeding not only have scientifically corroborated effects on health variables, but also on the cognitive and emotional development of children. A positive relationship of breastfeeding and performance in intelligence tests has been found in numerous studies that used a wide variety of intelligence tests and thus measured a variety of cognitive variables^6,7^.

There is also evidence of relationships of breastfeeding and psychological variables. A meta-analysis showed that children with attention-deficit/hyperactivity disorder (ADHD) were, on average, breastfed for a significantly shorter duration^8^. Breastfeeding for 12 to 24 months is also associated with a significantly lower risk of developing an autism spectrum disorder^9^. In addition to the positive effects on children, breastfeeding also has an impact on the mother. Women who breastfeed have a lower risk of developing breast or ovarian cancer^1^. They have decreased risks of, postnatal anxiety disorders and depression^10^, particularly in at risk mothers^10^, possibly because prolactin and oxytocin are released during breastfeeding^11^. Mothers who breastfed reported a stronger bond with their four-month-old infant than mothers who did not breastfeed^12^. Breastfeeding was also correlated with less negative affect and less intrusive behavior by the parents, and less child dysregulation^12^. While these results are based on findings from mothers breastfeeding one child at a time and ample studies exist on breastfeeding in general, research on tandem breastfeeding is still lacking. To the best of our knowledge, there are currently no quantitative studies or mixed methods studies on the psychology of tandem nursing. One qualitative study on tandem breastfeeding showed higher breast pain and sensitivity, insomnia and fatigue in tandem breastfeeding mothers^13^. As the literature is limited and focused on qualitative methods, the current set of studies aimed to set a foundation for psychological research on tandem breastfeeding using a wider variety of methods and more detailed reports. For this purpose, relevant topics were defined using a *qualitative* approach in Study 1 and their general applicability was tested in a *quantitative* approach in Study 2. This mixed methods design is particularly well suitable for the first mapping of such a novel topic of research^14^.

## Method study 1

All methods of both studies were performed in accordance with relevant guidelines and regulations, in particular the Declaration of Helsinki, and approved by the local ethics committee of the University of Bremen (ref. number: 2024-12). Informed consent was obtained from participants prior to their participation.

The subject of tandem breastfeeding is in a very rudimentary state^5^, with a major lack of research. An empirical basis is needed for a reliable, rather generalizing study. For this purpose, a qualitative study was carried out using interviews to generate hypotheses and themes for a later quantitative investigation.

### Sample

Two interviews were conducted with mothers who were tandem breastfeeding and two additional interviewed mothers had two children and also breastfed both children, but not in tandem. There was no financial compensation for the interviews. The interviewees were recruited through La Leche League breastfeeding support groups (for breastfeeding and for tandem breastfeeding mothers) in Germany.

### Process

The interviews were conducted between June 6, 2024 and June 13, 2024. Semi-structured, non-standardized interviews with open questions were conducted. The interviews were conducted online via Zoom X (video conferencing, web conferencing, online meetings, screen sharing).

### Evaluation

The transcribed interviews were evaluated using the MAXQDA program and a qualitative content analysis according to Mayring (MAXQDA, 22.4.1, VERBI^14,15^. For this purpose, using the transcribed interviews of the tandem breastfeeding mothers and suspected aspects related to tandem breastfeeding, a category system with coding rules was developed. This mainly inductive category system was iteratively expanded during the first two interviews and adapted during the evaluation process through inductive coding. Categories were redefined based on the subjective answers given by participants.

### Coding

The following is a list of the codes used as well as further explanations and prototypical examples for the codes. The behavior of the children after the birth of the younger child or after the start of tandem breastfeeding was coded. Below are two prototypical examples of the code *behavior of children after starting tandem breastfeeding*.

That was it, he was very clingy, very affectionate with his mother, and he was with us a lot. (Participant B, interview, June 7, 2024)

And during that time, yes, it’s easy to say, she developed into more like a daddy’s girl, who, when he came home from work in the evening, had a lot of time, unlike her mom, who she suddenly had to share all day. (Participant D, interview, June 13, 2024)

In addition, the categories of *fears* (referring to statements about fears related to breastfeeding or weaning)*, mother–child relationship* and *sibling’s relationship* were coded. In addition, we used the code *Parenting*. Reasons for tandem breastfeeding were coded with the code *Reasons for breastfeeding/tandem breastfeeding*, and all statements about pregnancy that did not refer to breastfeeding during pregnancy were coded with the code *Pregnancy*. The *cooperative skills* category refers to children’s cooperative and social skills and described children’s behaviors related to this topic, such as sharing. For example, this prototypical comment from a non-tandem breastfeeding mother.

I don’t know, we’re shopping or something and the little one says, I want a donut, then he would never take one just for himself, but he would always take one for his sister too. (Participant D, interview, June 13, 2024)

Furthermore, statements about the *environment* and *experiences* were also coded. The *environment* category was further divided into the subcodes *medical staff, social environment, partner and family*. This is about experiences with these people, as well as associations and feelings that were expressed in the interviews. In addition, the code *experience* was divided into seven subcodes: *experience of weaning, experience of breastfeeding the second child, experience of breastfeeding the first child, experience of breastfeeding in general, experience of breastfeeding the child, experience of tandem breastfeeding* and *experience of breastfeeding during pregnancy*. After conducting the qualitative content analysis, the coded elements were summarized and themes were generated for the questionnaire in Study 2.

## Method Study 2

### Sample

Based on the topics of Study 1, a questionnaire was designed for Study 2 to investigate relevant topics of tandem nursing in detail. Study 2 was preregistered with the Open Science Framework (10.17605/osf.io/ehngf). A total of 197 people took part in the survey, 93 of whom completed the survey. Three people were excluded because their children were older than the preregistered age range (10 years). In total data from 90 people were included in the analysis (Table 1). The sample was recruited using flyers and posts in (online) breastfeeding support groups, Facebook groups, and on Instagram. Flyers were also distributed in kindergartens, paediatricians’ offices, breastfeeding support centers, and at weekly markets in the Bremen and Oldenburg area. Parents were recruited via a local child study database. Furthermore, the study was promoted via the La Leche League newsletter and their homepage.

There was one diverse person in the control group and one diverse person in the group of tandem breastfeeding mothers, all other participants were female. Three people in the control group were single parents, while this only applied to one person in the group of tandem breastfeeding mothers. There were no relevant differences in the highest level of education between the tandem breastfeeding group and the control group among the participants themselves or among the participants’ partners. 

The average needs-weighted household net income was 2179.23 € (SD = 931.17, 95% CI [2374.26, 1984.2]). This information only contains data from 88 test subjects, as the information on monthly household net income for two people needed to be excluded due to being unrealistically high or low outliers. This average value is comparable to though slightly above the average value for Germany which is currently 2109 € (Institut der deutschen Wirtschaft (IW).). The average needs-weighted net household income of the control group (M = 2226.76, SD = 747.94, 95% CI [2459.84, 1993.69]) was slightly above the mean of the needs-weighted net household income of the tandem breastfeeding mothers (M = 2137.65, SD = 1072.45, 95%- CI [2449.05, 1826.24]). A total of 88 of the valid interviews were answered in the German-language form and 2 participants chose the English-language version. Most participants were from Germany, but there were also mothers from Great Britain (1), Austria (7) and Switzerland (2), all of whom were part of the tandem breastfeeding group. To participate, the test subjects were able to take part in a raffle for vouchers worth 50 euros.

In total, the data from 90 people, including 48 data sets from tandem breastfeeding mothers and 42 data sets from the control group, were included in the analyses. Some hypotheses could only be tested with a sub-set of the participants, as not all participants had a partner and not all participants had already weaned their older child.

**Table 1:** Gender of children.

Group		Male	Female	Diverse
Tandem group	Older children	43.75	54.17	2.08
	Younger children	50.00	50.00	0.00
Control group	Older children	40.48	59.52	0.00
	Younger children	59.52	40.48	0.00

Stated in percent.

It was noticeable that the older children in the control group were significantly older than the children who were breastfed in tandem (Table 2). A similar tendency was also evident among the younger siblings. Here too, the children from the control group were older than the younger siblings who were breastfed in tandem. However, it should also be noted that the average sibling distance between the children in the control group was significantly larger (M = 3.44 years, SD = 1.66, 95% CI [2.92, 3.95]) than in the tandem breastfeeding group (M = 2.35 years, SD = 1.05, 95% CI [2.04, 2.65]).

**Table 2:** Age of children.

Group		M	SD	CI
All	Older children	5.14	2.02	[4.72, 5.56]
	Younger children	2.28	1.72	[1.92, 2.64]
Tandem group	Older children	4.53	1.66	[4.05, 5.01]
	Younger children	2.18	1.59	[1.72, 2.64]
Control group	Older children	5.84	2.18	[5.15, 6.52]
	Younger children	2.4	1.86	[1.82, 2.98]

Stated in percent.

Overall, 67.78 percent of the participants had already weaned their older child; as expected, everyone in the control group had already weaned their older child; in the tandem breastfeeding group 39.8 percent. On average, the tandem-breastfed children were breastfed longer (M = 49.37 months, SD = 10.87, 95% CI [44.13, 54.61]) than the older children in the control group (M = 15.76 months, SD = 9.61, 95% CI [12.77, 18.76]). This was also evident for the duration of breastfeeding in the younger child. In the group of tandem breastfeeding mothers, the average duration of breastfeeding for the younger child was 34.25 months (SD = 18.23, 95% CI [19.00, 49.49]) and in the control group it was 15.7 months (SD = 11.68, 95% CI [ 10.23, 21.17]). The means for breastfeeding duration only include people who have already weaned their child. For the older children, 19 people from the tandem breastfeeding group and all people from the control group were included. For the younger child, data from eight tandem breastfeeding mothers and 20 mothers from the control group were included.

### Material

The findings from the qualitative content analysis were used in the creation of the questionnaire. The 16-item version of the PBQ was used to evaluate mother–child bonding^16^. The German translation of the SRQ-R was used to assess the sibling relationship^17^. Furthermore, the levels of authoritative, authoritarian and permissive parenting styles were measured using the Parenting Style Questionnaire^18^. The expression of attachment parenting style was assessed using a self-created question pool, including seven statements collected from the interviews and nine questions that emerged from research on the attachment parenting style, as well as 16 questions about the Baby B’s, which play a central role in attachment parenting^19^. The study was conducted online. Participants needed 32.1 min on average to complete the questionnaire.

### Study design

Analyses were conducted in line with the preregistration unless otherwise noted. For the analysis of the relationship between the nominally scaled variables, such as the type of breastfeeding, and the dependent variables, dependent and independent one-tailed t-tests as well as a mixed analysis of variance (Mixed ANOVA) were used. Furthermore, correlations were used to examine the connection between the duration of breastfeeding, and the dependent variables. Chi-square tests were used to assess the association between the nominally scaled variables. In order to prevent order effects, the questions regarding how pleasant breastfeeding was perceived were presented in a randomized order. Hypotheses were generated based on the qualitative analyses in Study 1 and preregistered with the Open Science Framework. An overview of all hypotheses and findings is presented in Table 3 (Supplement A).

## Results of Study 1

Through the interviews, we were able to identify five clusters that the mothers repeatedly addressed in their interviews. They led to the preregistered hypotheses for Study 2, stated in Table 3 (Supplement A).

**Cluster 1: relationships and bonding**. The mother–child bond in general, and problems in the relationship between the mother and, in particular, the older child, the topic of mother–child relationships was determined as relevant factors to be included in the questionnaire in Study 2. The tandem-breastfeeding mothers repeatedly reported that they had always been the older child’s primary caregiver since birth. Since the non-tandem-breastfeeding mothers in particular reported problems after the birth of the younger child, such as jealousy, rejection towards the mother, separation problems, difficulties in adjusting to the new family situation with a sibling, and a reorientation towards the partner of the mother, questions about this topic were included in the questionnaire. Since the mothers primarily associated these problems with the birth of their second child, the older child’s focus on the mother before and after the birth of the sibling was also added to the questionnaire. In addition, the sibling relationship was included in the questionnaire and assessed using the Sibling Relationship Questionnaire (SRQ-R)^17^, since tandem-breastfeeding mothers repeatedly mentioned that their children were very cautious with each other and sought physical closeness beyond the norm.

**Cluster 2: parenting**. Since mothers extensively mentioned actions and beliefs were addressed that can be attributed to, or contradict, different parenting styles, we decided to include a questionnaire about authoritarian, authoritative, and attachment parenting styles.

**Cluster 3: breastfeeding experience** / comparing experiences of tandem and non-tandem breastfeeding mothers. The mothers reported positive and negative emotions they associate with breastfeeding, but also reported pain while breastfeeding, aversion to breastfeeding, differences in how relaxed breastfeeding was perceived and how much the mothers felt their freedom was restricted by breastfeeding. The questionnaire therefore asked about how pleasant breastfeeding was, with the older child, with the younger child, and with both children together. The mothers also reported feelings of anger and being overwhelmed while breastfeeding, especially during pregnancy, with some of the anger also being directed at the older child. This was also included in the questionnaire.

The tandem-breastfeeding mothers reported being sick more often during pregnancy, which was also added to the questionnaire. Based on the interviews, we also asked how stressful the pregnancy with the younger child was experienced. The mothers were also asked whether they would breastfeed in the same way if they became pregnant again, because the interviews revealed different opinions about whether they would breastfeed again in the same way. The tandem-breastfeeding mothers reported that breastfeeding was physically strenuous, reflected in their general behavior and body weight. This topic was also addressed in the questionnaire. The question of whether they felt like sacrificing themselves for their children was also included based on statements made in the interviews by the tandem-breastfeeding mothers. Since sleep was repeatedly mentioned in the interviews, questions on this topic were also included.

Since all mothers repeatedly reported that they found breastfeeding practical and convenient, for example, when traveling, the questionnaire also asked about how practical breastfeeding was considered. The non-tandem-breastfeeding mothers reported feelings of guilt when weaning their older child and dissatisfaction with the duration of breastfeeding, so the topic of guilt regarding weaning was also included. The mothers also reported differences in how difficult weaning was, so this was also included. The interviews also revealed differences between tandem-breastfeeding and non-tandem-breastfeeding mothers regarding the openness with which they share their breastfeeding behavior with others and the reactions of those around them. Therefore, the questionnaire also asked whether the mothers felt that their breastfeeding behavior was accepted by those around them.

**Cluster 4: differences in the children’s social and cooperative skills**. The mothers addressed the topics of caring for others, sharing, jealousy, stubbornness, dealing with frustration, and cooperating in the face of criticism. The questionnaire therefore also assessed the children’s cooperative skills.

**Cluster 5: factors promoting tandem breastfeeding**. Mothers frequently discussed support from their partners, especially when putting the children to bed and also when bottle-feeding their children. Therefore, the questionnaire included the partners’ involvement. Tandem breastfeeding mothers also reported problems with their older child’s eating habits, such as refusing to eat puree or eating too little. Therefore, the questionnaire also addressed the eating habits of their older children. Tandem breastfeeding mothers also reported being discouraged from breastfeeding during pregnancy and receiving inadequate advice on tandem breastfeeding, so satisfaction with the advice on breastfeeding was also assessed. For the sake of completeness, we also examined whether there is a correlation between tandem breastfeeding and the duration of breastfeeding with socioeconomic status because one mother reported income as a possible variable for how long one can care for your own children at home.

### Results study 2

**Fig. 1:**
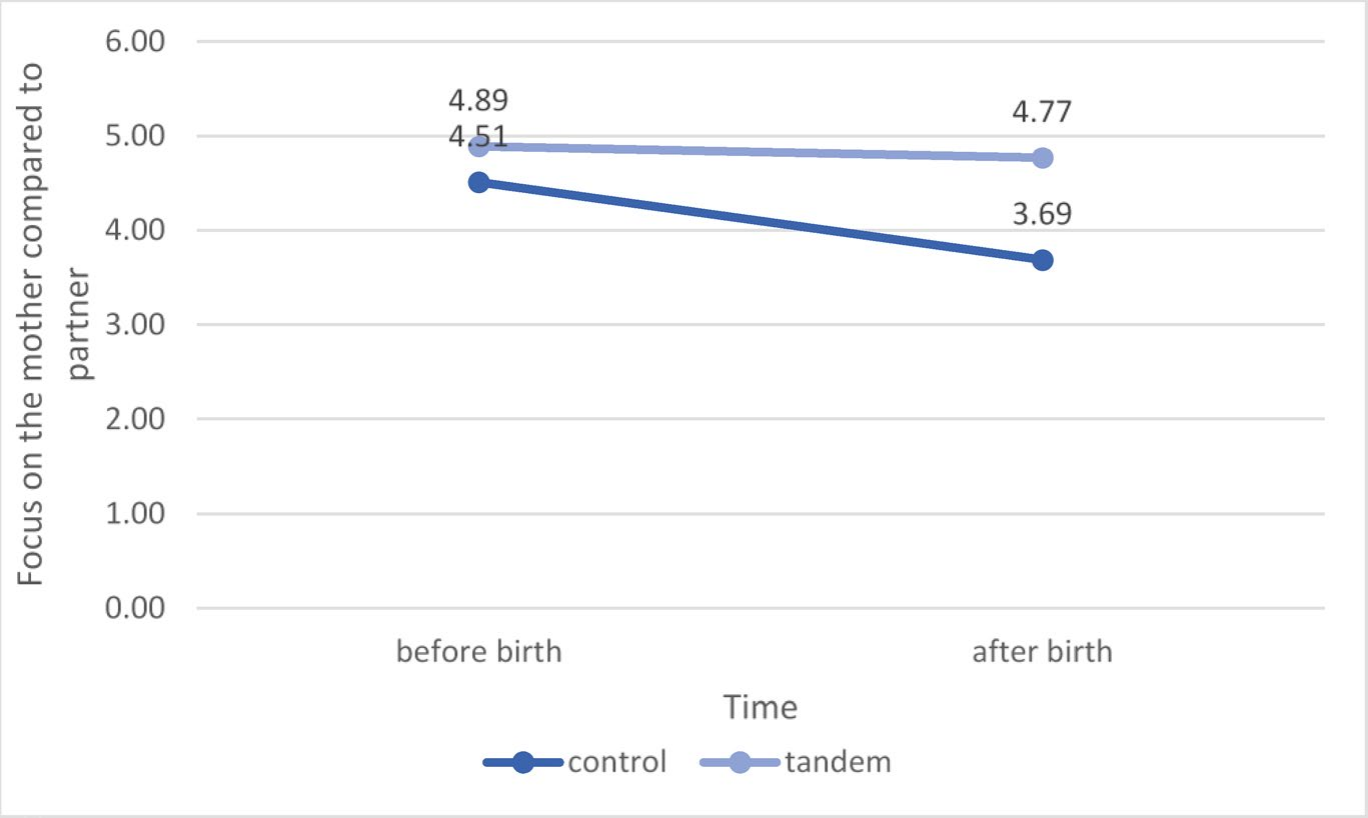
The interaction from time and group membership to the focus on the mothers or their partners. *Note.* Shown are the mean values ​​of the tandem breastfeeding mothers for the older children’s focus on the mothers before and after the birth of the younger child in dark blue and the mean values ​​of the control group for the older children’s focus on the mothers before and after the birth of the younger child in light blue.

The data from the quantitative study was analyzed using the R-Studio program with the Version 4.4.1^20^. There was a significant main effect of tandem breastfeeding on the older child’s focus on the mother, *F* (1,84) = 11.585, *p* = 0.001, *η*^*2*^ = 0.09, with the focus on the mother being greater in the older children breastfed in tandem (see Fig. 1). There was also a main effect for the focus on the mother before and after the birth of the younger sibling, *F* (1,84) = 12.676,* p* < 0.001, *η*^*2*^ = 0.04 (see Fig. 1). The interaction, as postulated in Hypothesis 2.1, was also significant,* F* (1,84) = 6.769,* p* = 0.011, *η*^*2*^ = 0.02 (see Fig. 1).

It was confirmed that the older siblings who were not breastfed in tandem were more focused on the partners than the tandem breastfed children and the difference was statistically significant, *t*(84) = -4.07, *p* < 0.001, *d* = -0.88, *BF* = 209,525.

Older siblings who were not breastfed in tandem were more focused on the partners after birth than before birth,* t* (38) = 4.48, *p* < 0.001,* d* = 0.72. Children who are not breastfed in tandem show more rejection towards their mothers after the birth of the younger child, *t* (74.51) = 2.10, *p* = 0.020, *d* = 0.45, *BF* = 1.622(Welch’s t-test). In tendency, the tandem breastfeeding mothers felt that their older children were on average clingier M = 3.56 than the older siblings in the control group M = 3.14. However, this result was not significant, *t* (88) = -1.63, *p* = 0.054, *d* = -0.34, *BF* = 0.701.

**Fig. 2:**
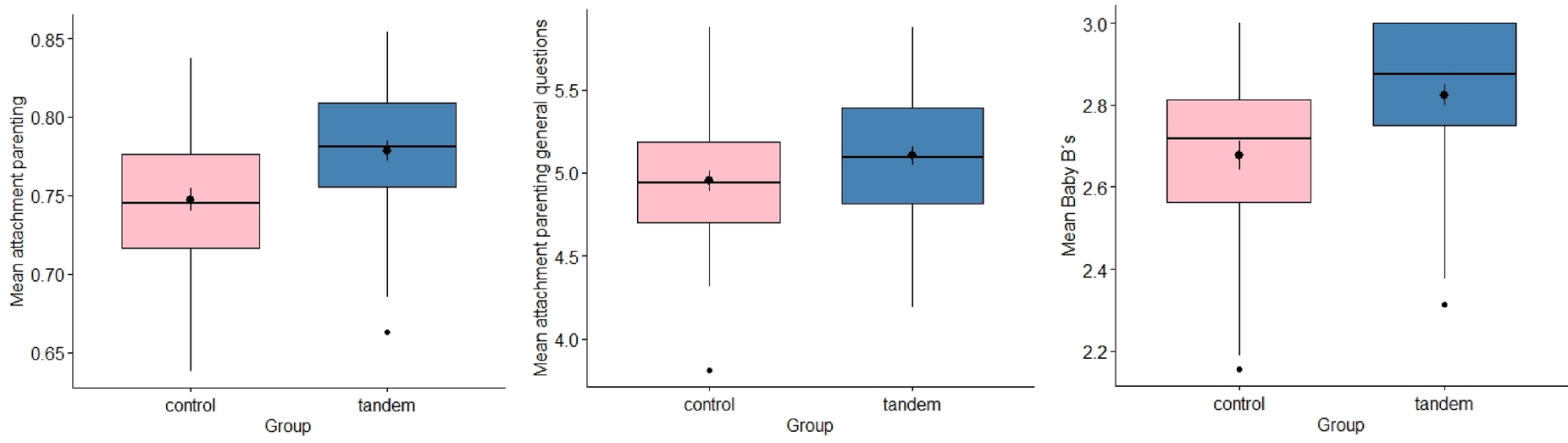
Differences in the expression of the attachment parenting style between the tandem breastfeeding mothers and the control group. *Note.* The differences in the expression of the attachment parenting style between the tandem breastfeeding mothers and the control group are displayed for the mean of the total questions (left), the general questions (middle) and the Baby B’s (right) using a box plot.

Tandem breastfeeding mothers received a lower average score in the questionnaire on authoritarian parenting, *t* (88) = 1.73, *p* = 0.044, *d* = 0.36, *BF* = 0.816 and higher attachment parenting style scores, *t* (88) = -3.28, *p* = 0.001, *d* = -0.69, *BF* ​​ = 21.118, Fig. 2.

An additional exploratory analysis, separately including only the general items in the attachment parenting questionnaire (*t* (88) = -1.82, *p* = 0.036, *d* = -0.38, *BF* = 0.934) and only the Baby B’s (*t* (88) =− 3.47, *p* < 0.001, *d* = -0.73, *BF* = 35.708) showed significant differences (see Fig. 2).

Tandem breastfeeding mothers find breastfeeding the older child after the birth of the younger child to be more unpleasant than breastfeeding the younger child, *t*(47) = 7.25, *p* < 0.001, *d* = 1.05. In addition, tandem breastfeeding mothers find breastfeeding the older child before the birth of the sibling to be more pleasant than afterwards, *t* (47) = 3.36, *p* = 0.001, *d* = 0.48. The hypothesis that the mothers in the control group would prefer to breastfeed another child in the same way was tested with a Welch’s t-test and can also be rejected based on the data. There was a tendency in the opposite direction, *t* (58.10) = -2.44, *p* = 0.991, *d* = -0.53, *BF* = 3.568. An exploratory two-tailed t-test was then calculated, which showed that this difference between the groups was significant, *t* (58.10) = -2.44, *p* = 0.018, *d* = -0.53, *BF* = 3.568.

**Fig. 3:**
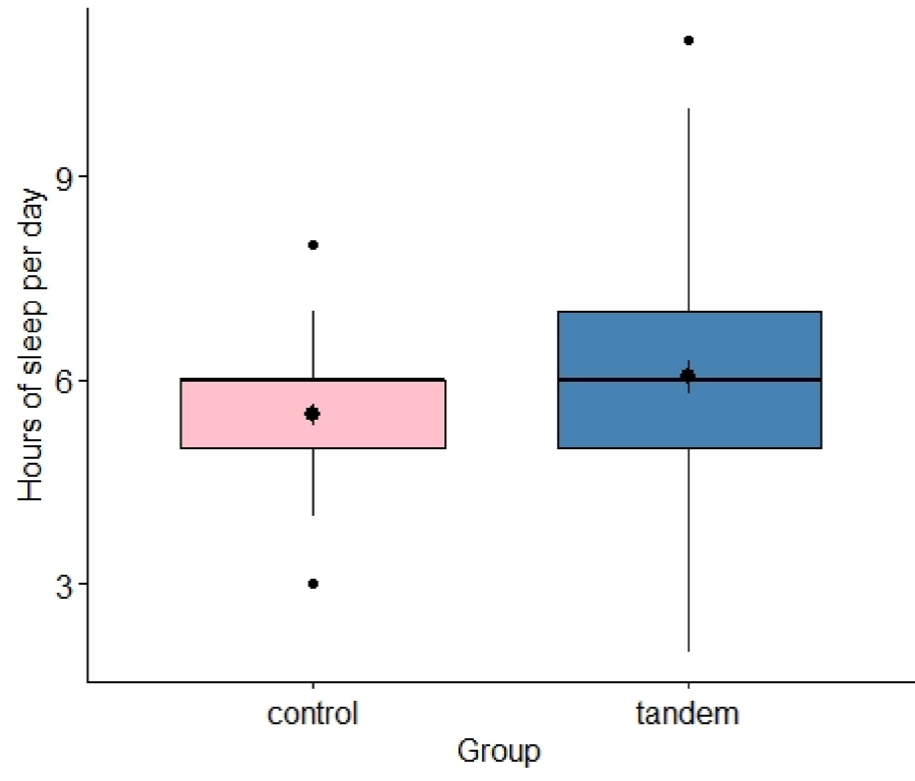
Differences in the hours slept per day after the birth of the younger child between the tandem breastfeeding mothers and the control group. *Note.* The differences in the hours the mother slept per day after the birth of the younger child between the tandem breastfeeding mothers and the control group are shown using a box plot.

Tandem breastfeeding mothers found breastfeeding two children more energy-consuming than breastfeeding one child, *t*(47) = -3.31, *p* = 0.001, *d* = -0.48. They experience breastfeeding as more practical, *t*(61.84) = -2.27, *p* = 0.013, *d* = -0.49, *BF* ​​ = 2.411. The hypothesis that tandem breastfeeding mothers feel more tired after the birth of their younger child could not be confirmed. The tests showed a trend in the opposite direction, *t*(88) = -1.78, *p* = 0.960, *d* = -0.37, *BF* = 0.874. The average hours of sleep showed a similar pattern, *t*(88) = -1.85, *p* = 0.966, *d* = -0.39, *BF* = 0.981, Fig. 3.

**Fig. 4:**
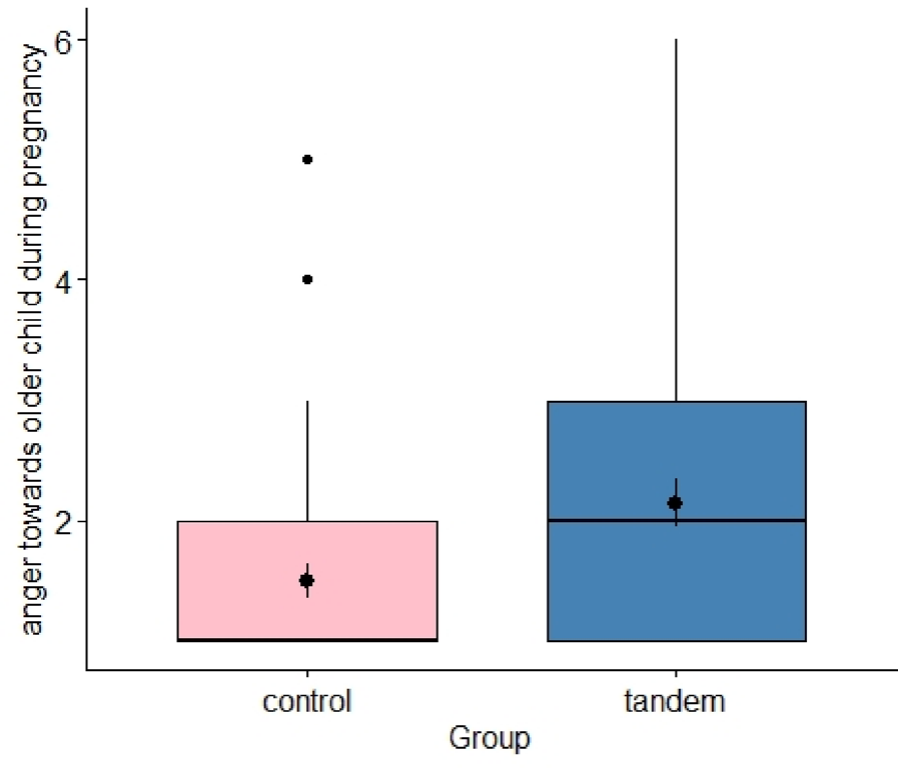
Anger and rage towards the older child after the birth of the sibling in a comparison between the tandem breastfeeding mothers and the control group.

With regard to the increased anger and feelings of aggression towards the older child during pregnancy with the sibling in tandem breastfeeding mothers, a significant difference can be found between the groups, *t*(81.48) = -2.60, *p* = 0.006, *d* = -0.54, *BF* = 3.496, 4.

**Fig. 5:**
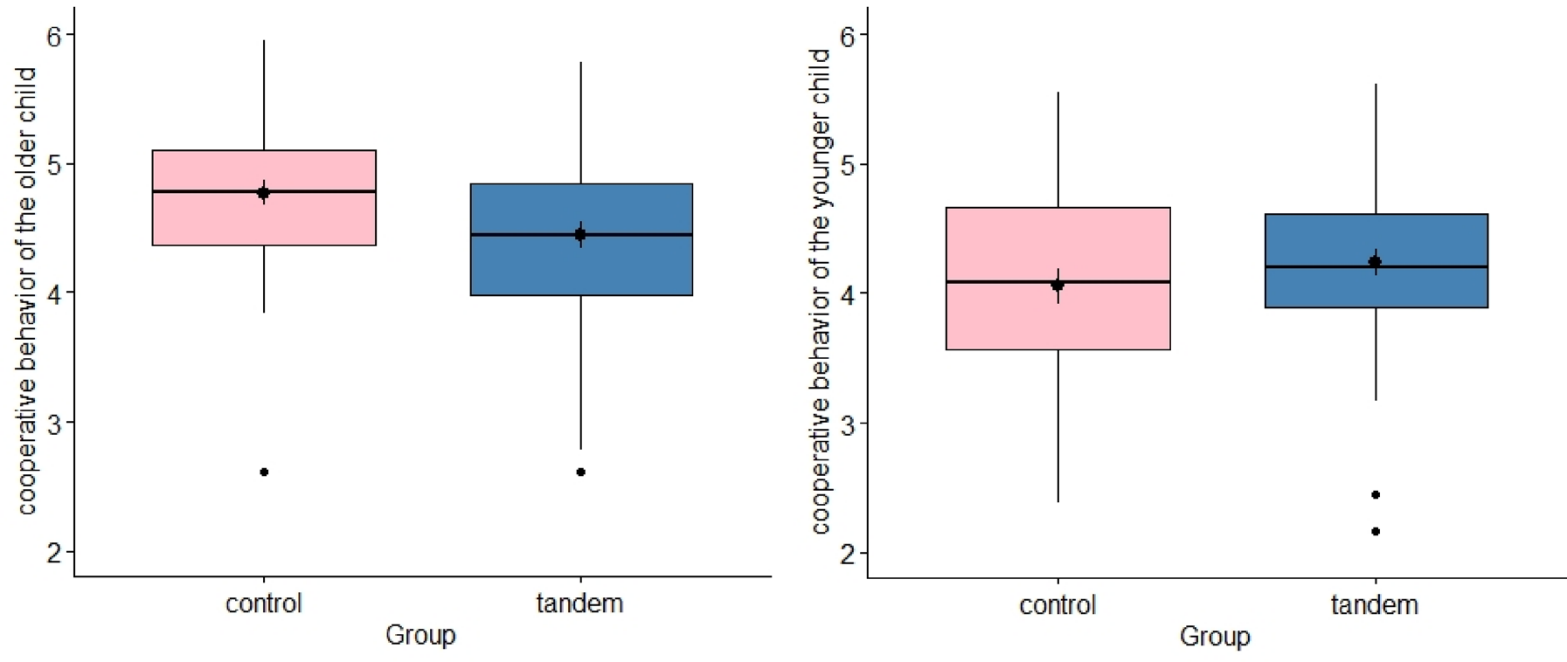
Differences in children’s cooperative behavior for younger and older child.

Tandem breastfeeding mothers have the feeling that their breastfeeding behavior is less accepted, *t* (88) = 5.36, *p* < 0.001, *d* = 1.13, *BF* = 20,297.12. The hypothesis that tandem-breastfed children show more cooperative behavior was not confirmed, *t* (88) = 0.56, *p* = 0.713, *d* = 0.12, *BF* = 0.255. This also applied when we only considering the younger child in an exploratory analysis, *t*(88) = -1.13, *p* = 0.261, *d* = -0.24, *BF* = 0.388 (see Fig. 5). However, when an exploratory analysis was carried out only with the data of the older children, a difference emerged in the opposite direction, with the non-tandem breastfeeding mothers reporting more cooperative behavior, *t*(88) = 2.31, *p* = 0.023, *d* = 0.49, *BF* = 2.249 (see Fig. 5).

**Fig. 6:**
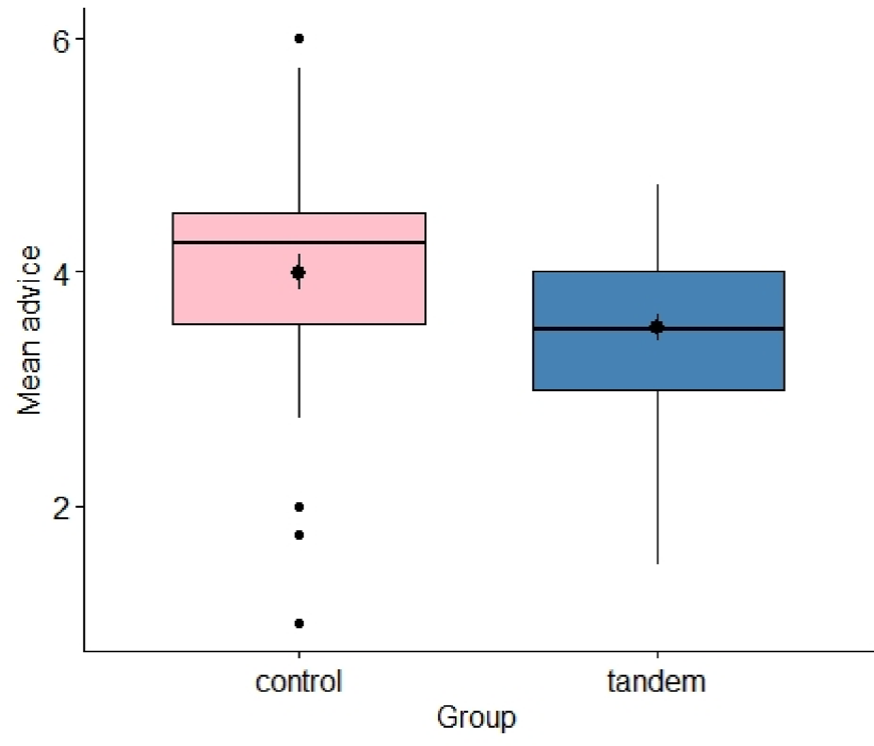
Differences in how well the mothers felt advised about breastfeeding. *Note*. The differences in how well the mothers felt advised about breastfeeding between the tandem breastfeeding mothers and the control group are shown using a box plot.

The hypothesis that tandem breastfeeding mothers feel less well advised about breastfeeding was confirmed, *t*(88) = 2.51, *p* = 0.007, *d* = 0.53, *BF* = 3.377 (see Fig. 6). The results of all hypotheses from the pre-registration are shown in Table 3 (see Supplement A).

## Discussion study 1 and 2

The current set of studies used a mixed design to firstly get an overview of psychological factors involved in tandem nursing in the qualitative study 1 and subsequently test the identified factors in a larger quantitative study 2.

In study 1, the interviews with the coded elements and their analysis provided insights into the following topics: mother–child bonding, sibling relationships, parenting style, children’s cooperative behavior, children’s jealousy and reactions to the birth of a sibling, experiences with breastfeeding and weaning, the mother’s sleep patterns, the children’s eating habits, the partner’s behavior and opinions, social criticism, and counseling. Since the categories *experiences, environment, reasons for breastfeeding/tandem breastfeeding, cooperative skills, siblings’ relationship* and *mother–child relationship* were particularly frequently assigned, these topics were addressed with more questions in the questionnaire for Study 2. These included questions regarding mother–child bonding, focus on mother/partner, sibling relationship, sleep, weaning of the older child, cooperative skills of the children, the ability of children to share, eating behavior of the older child, behavior of the partner, how pleasant or practical breastfeeding is experienced and what breastfeeding behavior would look like with another child in the future. The topic of parenting was also chosen as a broader theme in the questionnaire, as the coded elements related to parenting contained indications of various parenting styles, which were then individually addressed in the questionnaire. Since the coded elements from tandem-breastfeeding mothers regarding the *environment* could predominantly be assigned to the subcodes *partner and family*, as well as *social environment*, these two topics were included in the questionnaire, while the topic of *medical staff* was not.

Study 2 quantitatively investigated the topics of relevance regarding tandem nursing that were identified in Study 1. Some significant differences between tandem and non-tandem nursing groups were confirmed. Children who were breastfed in tandem were more focused on their mothers and there was no significant difference between their focus before the birth of the sibling or after. In contrast, the children in the control group were generally less focused on the mothers and this also reduced after the birth of the sibling. The older siblings who were not breastfed in tandem rejected the mothers more after the birth of the younger child than the children who were breastfed in tandem. Tandem-breastfeeding was associated with mothers’ higher scores on attachment parenting. There is also a psychological shift in tandem-breastfeeding mothers: before birth they perceive the breastfeeding of their older child as more pleasant than after birth and after birth, they find breastfeeding the younger child more pleasant than the older child, have more anger towards the older child during pregnancy and they find breastfeeding two children more energy-consuming than one child. Tandem-breastfeeding mothers found breastfeeding more practical than non-tandem-breastfeeding mothers. Tandem-breastfeeding mothers feel less well informed about their type of breastfeeding and less accepted by society.

The development that the older child becomes less attached to the mother after the birth of a sibling has been confirmed in other studies^21^. Tandem breastfeeding thus counteracts the effect that the older children focus more on the mothers’ partners after the birth of their siblings. This development can sometimes be painful for the mothers and also challenges and restructures the family system. Overall, older siblings seem to be more focused on the mother when she tandem breastfeeds than when she does not. Therefore, the mother will also need to invest more resources and capacity in the older child when tandem breastfeeding. It is therefore particularly important that tandem breastfeeding mothers are supported by their partners and a support system, so that they can be relieved of their heightened care-workload. This is particularly relevant as negative feelings towards the older child seemed to be increased in tandem nursing mothers. This may be related to the general high load of care work, in which case a support system is crucial.

On average, the tandem breastfeeding mothers received significantly higher scores of attachment parenting style. This difference could be explained by the fact that the attachment parenting style also recommends that children should wean themselves when they are ready^22^. However, the natural weaning age is two and a half to seven years^23^. The current trends towards attachment parenting can be one reason why, due to the longer duration of breastfeeding, tandem breastfeeding increases.

When it came to general bonding with the mother in the first two weeks after birth, there was no overall connection with tandem breastfeeding for all subtests. However, there may be a floor effect, since a lower value represents a stronger mother–child bond. All mothers breastfed their children after birth. As breastfeeding in general has a positive influence on the bond with the infant, especially in the first months after birth^12^, which was assessed using the PBQ^16^, tandem breastfeeding might not further increase the positive effect of breastfeeding on the mother–child bond. There was also no connection with tandem breastfeeding when it came to adapting to the new sibling.

The third block of hypotheses addressed the experience of breastfeeding and weaning, as well as pregnancy. Tandem breastfeeding mothers found breastfeeding the older child after the birth of the younger child more unpleasant than breastfeeding the younger child or the older child before the sibling is born. It cannot be determined from the data whether this perception is due to tandem breastfeeding or is due to the age of the child and the longer duration of breastfeeding. However, in a large study from Australia, women who were tandem breastfeeding also experienced breastfeeding aversion more often when they started tandem breastfeeding^24^. In addition, many women have an aversion to breastfeeding for the first time when the child is a toddler^24^. However, it was also found that tandem breastfeeding mothers would more likely prefer to breastfeed again in the same way, if they had another child, than non-tandem breastfeeding mothers.

Regarding sleep, there was a non-significant tendency for tandem breastfeeding mothers to feel more rested and to have more hours of sleep per day after the birth of the younger child, although an opposite result was assumed^25^. A qualitative study on tandem breastfeeding reported that tandem breastfeeding mothers would have problems with insomnia and fatigue^13^. The current study contradicts these findings. A possible explanation it that breastfeeding hormones increase sleep quality in mothers^11,26,27^ and children^28^, with hormones having a stronger influence in tandem breastfeeding^29^.

Tandem breastfeeding mothers felt significantly more anger towards their older child while being pregnant with the younger child than the control group. From an evolutionary perspective, an aversion towards the older child during breastfeeding may ensure that the smaller child receives a sufficient amount of breastmilk and nutrients. Therefore, one speculation might be that greater anger towards the older child may decrease breastfeeding frequency, securing resources for the younger child. Nevertheless, the tandem breastfeeding mothers did not experience the pregnancy as more stressful than the control group. But the mothers who breastfeed in tandem usually also breastfeed during pregnancy and therefore increased prolactin is released during pregnancy, which may help with stress modulation^30^. Alternatively, breastfeeding may be an additional task for the mother during pregnancy, increasing her workload and therefore resulting in overwhelm. Future work should study if anger is reduced with a fully functioning support system compared to lack of support for the mother.

As expected, the tandem breastfeeding mothers reported less acceptance for their breastfeeding behavior. This could be related to the fact that tandem breastfeeding is less widespread. In addition, social criticism of breastfeeding increases with the age of the children^31^ and the breastfeeding duration of the tandem breastfeeding children was significantly longer than in the control group. It is possible that the negative judgement from outside promotes breastfeeding aversion and negative feelings towards the older child in tandem nursing mothers, which should be further investigated. If family and friends support the breastfeeding behavior, that supports a longer duration of breastfeeding^32^. With recent developments promoting breastfeeding, more recent research on current acceptance of breastfeeding is necessary.

The children showed no significant difference in their cooperative behavior and ability to share. The only exception is the cooperative behavior of the older children, as the children in the control group showed significantly more cooperative behavior. The opposite result was expected, as the risk of child behavior disorders decreases as the duration of breastfeeding increases^33^. Furthermore, previous beliefs were that tandem breastfeeding leads to good interactions between siblings due to the increased physical proximity and the inclusion of the older child in breastfeeding, and that the older child thus increasingly takes on helping qualities and a caring sibling role^34^. The average age of the older siblings in the control group was 5.84 years and in the tandem breastfeeding group it was 4.53 years. Prosocial behaviors such as helping, comforting and sharing begin to develop in the second year of life^35^. However, understanding other perspectives develops between the ages of three and five^36^, as does responding to their friends’ wishes and sharing toys^35^. Therefore, the age difference of the older siblings in the groups could have confounded the results. This finding shows an important potential natural confound: siblings who are closer together in age are more likely to be tandem breastfed and therefore the socio-emotional development of the older sibling will be less advanced at the birth of their younger sibling that it would be if this child was older.

Tandem breastfeeding mothers felt less well advised about breastfeeding than the control group. These statements from the tandem breastfeeding mothers probably also refer to the quality of advice on tandem breastfeeding and this could be related to the rudimentary study situation^5^.

### Implications

The current major shortage of research on the subject of tandem breastfeeding is mainly related to medical aspects and only very few studies deal with individual psychological aspects. This study, which examines a large variety of topics connected to tandem breastfeeding, provides insights into which topics future research should focus on. It was found that tandem breastfeeding mothers felt less well advised about breastfeeding. This could be related to the fact that little research has been published on the topic of tandem breastfeeding and therefore healthcare professionals have no scientific sources on which they can base their advice, underlining the importance research on tandem breastfeeding. Furthermore, tandem breastfeeding was associated with negative emotions, which may be related to mothers being overworked with care responsibilities, as breastfeeding is mainly possible for females. Current developments show that fathers are increasingly involved in caretaking. Taken together with our findings, fathers should be encouraged to support their female partners in any possible way to decrease the workload associated with tandem breastfeeding.

## Conclusion

The current set of studies qualitatively generated relevant themes and quantitatively investigated psychological aspects of tandem nursing. Numerous potential differences between tandem nursing and non-tandem nursing were identified in the qualitative Study 1. However, only some of them were confirmed in the larger sample of Study 2. Specifically, the child’s focus on the mother differs: Tandem-breastfed older siblings were more focused on their mothers, with no differences before and after the birth of the sibling; in contrast, the children in the control group were less focused on their mothers overall, and this tendency became even clearer after the birth of the sibling. In addition, the children who were not breastfed in tandem rejected their mothers more after the birth of their sibling. In contrast, the mothers felt more anger towards the older child when tandem-nursing. The current set of studies shows that more research is needed on the topic of tandem nursing, as tandem nursing parents lack information and may require help, for example as they are more likely to find breastfeeding unpleasant or even have an aversion to breastfeeding und therefore should receive sufficient support. The large variety of topics investigated in the current set of studies provides an indispensable starting ground for future research and a first map of the psychology of tandem nursing.

## Supplementary Information

Below is the link to the electronic supplementary material.

Supplementary Material 1

## Data Availability

The data that support the findings of this study are openly available on the Open Science Framework (https://osf.io/m6s9c/files/osfstorage).

## References

[1] World Health Organization: WHO. (2019, November 11). Breastfeeding. https://www.who.int/health-topics/breastfeeding#tab=tab_1. Last access 28 Aug 2024.

[2] Bryant, T. Tandem nursing: A review and guidelines. *Int. J. Childbirth Educ. 27*(4) (2012).

[3] Yngve, A. & Sjöström, M. Breastfeeding in countries of the European Union and EFTA: Current and proposed recommendations, rationale, prevalence, duration and trends. *Public Health Nutr.* **4**(2b), 631–645. 10.1079/PHN2001147 (2001). 11683556 10.1079/phn2001147

[4] Brettschneider, A. K., von der Lippe, E. & Lange, C. Stillverhalten in Deutschland–Neues aus KiGGS Welle 2. *Bundesgesundheitsbl. Gesundheitsforsch. Gesundheitsschutz*10.1007/s00103-018-2770-7 (2018). 10.1007/s00103-018-2770-7 29934682

[5] Hanáčková, V., & Masopustová, Z. Tandem nursing as specific means of attachment parenting–mothers’ experiences. *E-psychologie* (2021). 10.29364/epsy.419

[6] Boutwell, B. B., Young, J. T. & Meldrum, R. C. On the positive relationship between breastfeeding & intelligence. *Dev. Psychol.* **54**(8), 1426. 10.1037/dev0000537 (2018). 29952603 10.1037/dev0000537

[7] Kim, K. M. & Choi, J. W. Associations between breastfeeding and cognitive function in children from early childhood to school age: A prospective birth cohort study. *Int. Breastfeed. J.* **15**, 1–9. 10.1186/s13006-020-00326-4 (2020). 32993704 10.1186/s13006-020-00326-4 PMC7526146

[8] Tseng, P. T. et al. Maternal breastfeeding and attention-deficit/hyperactivity disorder in children: a meta-analysis. *Eur. Child Adolescent Psychiatry* **28**, 19–30. 10.1007/s00787-018-1182-4 (2019). 10.1007/s00787-018-1182-4 29907910

[9] Ghozy, S. et al. Association of breastfeeding status with risk of autism spectrum disorder: A systematic review, dose-response analysis and meta-analysis. *Asian J. Psychiatr.* **48**, 101916. 10.1016/j.ajp.2019.101916 (2020). 31923810 10.1016/j.ajp.2019.101916

[10] Ystrom, E. Breastfeeding cessation and symptoms of anxiety and depression: A longitudinal cohort study. *BMC Pregnancy Childbirth* **12**, 1–6. 10.1186/1471-2393-12-36 (2012). 22621668 10.1186/1471-2393-12-36 PMC3449190

[11] Sibolboro Mezzacappa, E. & Endicott, J. Parity mediates the association between infant feeding method and maternal depressive symptoms in the postpartum. *Arch. Womens Ment. Health* **10**, 259–266 (2007). 18040595 10.1007/s00737-007-0207-7

[12] Else-Quest, N. M., Hyde, J. S. & Clark, R. Breastfeeding, bonding, and the mother-infant relationship. *Merrill-Palmer Q.*10.1353/mpq.2003.0020 (2003).

[13] Aker, M. N., Gönenç, I. M., Korucu, A. E. & Çalbayram, N. Ç. Mothers’ experiences of tandem breastfeeding: A phenomenological study. *Am. J. Perinatol.* **41**(S 01), e1421–e1434. 10.1055/a-2033-0031 (2024). 36764329 10.1055/a-2033-0031

[14] Mayring, P. & Fenzl, T. *Qualitative inhaltsanalyse* pp. 633–648 (Springer Fachmedien Wiesbaden, 2019).

[15] VERBI Software. *MAXQDA 2022 [computer software]* (VERBI Software, 2021). Available from maxqda.com.

[16] Reck, C. et al. The German version of the postpartum bonding instrument: Psychometric properties and association with postpartum depression. *Arch. Womens Ment. Health.* **9**, 265–271. 10.1007/s00737-006-0144-x (2006). 16937316 10.1007/s00737-006-0144-x

[17] Bojanowski, S. *Geschwisterbeziehungen im Kontext psychischer Erkrankungen* (Doctoral dissertation, Universität Potsdam, 2016).

[18] Robinson, C. C., Mandleco, B., Olsen, S. F. & Hart, C. H. Authoritative, authoritarian, and permissive parenting practices: Development of a new measure. *Psychol. Rep.* **77**(3), 819–830. 10.2466/pr0.1995.77.3.819 (1995).

[19] Sears, W., & Sears, M. *The attachment parenting book: A commonsense guide to understanding and nurturing your baby*. (Hachette UK, 2001).

[20] R Core Team. *R: A Language and Environment for Statistical Computing* (R Foundation for Statistical Computing, 2021).

[21] Volling, B. L. Family transitions following the birth of a sibling: An empirical review of changes in the firstborn’s adjustment. *Psychol. Bull.* **138**(3), 497. 10.1037/a0026921 (2012). 22289107 10.1037/a0026921 PMC3341504

[22] Miller, P. M. & Commons, M. L. The benefits of attachment parenting for infants and children: A behavioral developmental view. *Behav. Dev. Bull.* **16**(1), 1. 10.1037/h0100514 (2010).

[23] Dettwyler, K. A natural age of weaning. In *Brief Version of the Chapter “A Time to Wean,” in Breastfeeding: Biocultural Perspectives, edited by Patricia Stuart-Macadam and Katherine A. Dettwyler*, 39–73 (1999).

[24] Morns, M. A., Burns, E., McIntyre, E. & Steel, A. E. The prevalence of breastfeeding aversion response in Australia: A national cross-sectional survey. *Matern. Child Nutr.* **19**(4), 13536. 10.1111/mcn.13536 (2023). 10.1111/mcn.13536 PMC10483935 37226968

[25] Galbally, M., Lewis, A. J., McEgan, K., Scalzo, K. & Islam, F. A. Breastfeeding and infant sleep patterns: An Australian population study. *J. Paediatr. Child Health* **49**(2), 147–152. 10.1111/jpc.12089 (2013). 10.1111/jpc.12089 23331519

[26] Huang, S. K. & Chih, M. H. Increased breastfeeding frequency enhances milk production and infant weight gain: Correlation with the basal maternal prolactin level. *Breastfeed. Med.* **15**(10), 639–645. 10.1089/bfm.2020.0024 (2020). 32799538 10.1089/bfm.2020.0024

[27] Roky, R. et al. Prolactin and rapid eye movement sleep regulation. *Sleep* **18**(7), 536–542. 10.1093/sleep/18.7.536 (1995). 8552923

[28] Cohen Engler, A., Hadash, A., Shehadeh, N. & Pillar, G. Breastfeeding may improve nocturnal sleep and reduce infantile colic: Potential role of breast milk melatonin. *Eur. J. Pediatr.* **171**, 729–732. 10.1007/s00431-011-1659-3 (2012). 22205210 10.1007/s00431-011-1659-3

[29] Blyton, D. M., Sullivan, C. E. & Edwards, N. Lactation is associated with an increase in slow-wave sleep in women. *J. Sleep Res.* **11**(4), 297–303. 10.1046/j.1365-2869.2002.00315.x (2002). 12464097 10.1046/j.1365-2869.2002.00315.x

[30] Faron-Górecka, A. et al. The involvement of prolactin in stress-related disorders. *Int. J. Environ. Res. Public Health* **20**(4), 3257. 10.3390/ijerph20043257 (2023). 36833950 10.3390/ijerph20043257 PMC9959798

[31] Kendall-Tackett, K. A. & Sugarman, M. The social consequences of long-term breastfeeding. *J. Hum. Lact.* **11**(3), 179–183. 10.1177/089033449501100316 (1995). 7669236 10.1177/089033449501100316

[32] Faleiros, F. T. V., Trezza, E. M. C. & Carandina, L. Factors influencing breastfeeding decision and duration. *Rev. Nutr.* **19**(5), 623–630. 10.1590/S1415-52732006000500010 (2006).

[33] Poton, W. L., Soares, A. L. G., Oliveira, E. R. A. D. & Gonçalves, H. Breastfeeding and behavior disorders among children and adolescents: A systematic review. *Rev. Saude Publica* **52**, 9. 10.11606/S1518-8787.2018052000439 (2018). 29412376 10.11606/S1518-8787.2018052000439 PMC5802715

[34] Flower, H. *Adventures in Tandem Nursing: Breastfeeding During Pregnancy And Beyond* Vol. 2 (Hilary Flower (self-published), 2019).

[35] Schneider, W. & Lindenberger, U. *Entwicklungspsychologie* (Beltz, 2018).

[36] Wimmer, H. & Perner, J. Beliefs about beliefs: Representation and constraining function of wrong beliefs in young children’s understanding of deception. *Cognition* **13**(1), 103–128. 10.1016/0010-0277(83)90004-5 (1983). 6681741 10.1016/0010-0277(83)90004-5

[37] *Geburtenfolge und Geburtenabstand*. (2024). Statistisches Bundesamt. Retrieved from https://www.destatis.de/DE/Themen/Gesellschaft-Umwelt/Bevoelkerung/Geburten/Tabellen/_tabellen-innen-geburtenfolge.html. Last access 28 Aug 2024

[38] Video conferencing, web conferencing, online meetings, screen sharing - Zoom. (n.d.). Retrieved from https://uni-bremen.zoom.us/

[39] Institut der deutschen Wirtschaft (IW) (2025). *Wie wohlhabend bin ich im Vergleich?* iwkoeln.de. Retrieved from https://www.iwkoeln.de/fileadmin/user_upload/HTML/2022/Einkommensrechner/index.html. Last access 24 Feb 2025.

