# Supplementary material for: Psychological relation of tandem nursing to children’s socio-emotional development and attachment to their caregiver
Source: Sci Rep. 2026 May 19;16:15461. doi: 10.1038/s41598-026-52462-2 (PMC13186955; doi:10.1038/s41598-026-52462-2)
Supplement: Supplementary file 1 — Supplementary Material 1 [file 41598_2026_52462_MOESM1_ESM.docx]

Supplement A

Table 3

*Summary of results*

| **Hypothesis** | **Result** | **Mean tandemgroup**  **(standard deviation)** | **Mean controlgroup**  **(standard deviation)** |
| --- | --- | --- | --- |
| 1.1. Siblings who are breastfed in tandem are more gentle with each other than siblings who are not breastfed in tandem. | *t*(88) = 1.72, *p* = .956, *d* = 0.36, *BF* =0 .805 | 3.04 (1.22) | 3.45 (1.02) |
| 1.2. Siblings who are breastfed in tandem show more physical closeness to each other than siblings who are not breastfed in tandem. | *t*(88) = 1.80, *p* = .962, *d* = 0.38, *BF* = 0.902 | 3.86 (1.06) | 4.23 (0.82) |
| 1.3. Siblings who are breastfed in tandem have a better relationship with each other than siblings who are not breastfed in tandem. | *t*(88) = 1.49, *p* = .930, *d* = 0.31, *BF* = 0.583 (warmth/closeness);  *t*(88) = 0.53, *p* = .300, *d* = 0.11, *BF* = 0.250 (relative status/power);  *t*(88) = -0.01, *p* = .503, *d* = 0.00, *BF* = 0.221  (conflict);  *t*(88) = -0.55, *p* = .709, *d* = -0.12, *BF* =0.253 (rivalry) | 3.59 (0.59)  7.46 (4.82)  2.64 (0.84)  2.97 (0.3) | 3.78 (0.58)  8 (4.91)  2.63 (0.95)  2.93 (0.32) |
| 2.1. There is an interaction between the focus on the mother or on the partner before and after birth and the tandem-breastfeeding | *F* (1,84) = 11.585, *p* = .001, *η²* = 0.09 (group);  *F* (1,84) = 12.676, *p* < .001, *η²* = 0.04 (time);  *F* (1,84) = 6.769, *p* = .011, *η²* = 0.02 (interaction) | 4.89 (1.07) before birth  4.77 (1.27)  after birth | 4.51 (1.14)  before birth  3.69 (1.15)  after birth |
| 2.2. Older children who were breastfed in tandem are more focused on their mother and less focused on the partner of their mother after the birth of the younger child than children who were not breastfed in tandem. | *t*(84) = -4.07, *p* < .001, *d* = -0.88, *BF* = 209.525 | 4.77 (1.27)  after birth | 3.69 (1.15)  after birth |
| 2.3. Older children who were not breastfed in tandem are more focused on the partner of their mother and less focused on their mother after the birth of the younger child than children who were breastfed in tandem. | *t*(84) = -4.07, *p* < .001, *d* = -0.88, *BF* = 209.525 | 4.77 (1.27)  after birth | 3.69 (1.15)  after birth |
| 2.4. Older children who were not breastfed in tandem are more focused on the partner of their mother and less focused on their mother after the birth of the younger child than before the birth of the younger child. | *t*(38) = 4.48, *p* < .001, *d* = 0.72 |  | 4.51 (1.14)  before birth  3.69 (1.15)  after birth |
| 2.5. Older children who were breastfed in tandem are more focused on their mother and less focused on the partner of their mother after the birth of the younger child than before the birth of the younger child. | *t*(46) = 0.68, *p* = .749, *d* = 0.10 | 4.89 (1.07)  before birth  4.77 (1.27)  after birth |  |
| 2.6. Children who were breastfed in tandem show stronger bonding with their mother than children who were not breastfed in tandem. | *t*(88) = 0.32, *p* = .375, *d* = 0.07, *BF* = .231 | 22.62 (14.03) | 23.57 (13.98) |
| 2.7. Older children who are breastfed in tandem are less jealous after the birth of the younger child than children who are not breastfed in tandem. | *t*(88) = 0.18, *p* = .428, *d* = 0.04, *BF* = 0.224 | 3.51 (1.34) | 3.56 (1.18) |
| 2.8. Older children who are not breastfed in tandem show more jealousy when the younger child is breastfed than children who are breastfed in tandem. | *t*(88) = -0.76, *p* = .776, *d* = -0.16, *BF* = 0.286. | 2.65 (1.29) | 2.44 (1.26) |
| 2.9. Older children who are not tandem breastfed show more rejection towards their mother after the birth of the younger child than children who are tandem breastfed. | *t*(74.51) = 2.10, *p* = .020, *d* = 0.45, *BF* = 1.622 | 1.83 (0.98) | 2.36 (1.33) |
| 2.10. Older children who are breastfed in tandem adapt to the new situation with a sibling more quickly than children who are not breastfed in tandem. | t(88) = -0.10, *p* = .461, *d* = -0.02, *BF* = 0.222 | 4.65 (1.33) | 4.62 (1.27) |
| 2.11. Older children who are breastfed in tandem are clingier towards their mother after the birth of the younger child than children who are not breastfed in tandem. | *t*(88) = -1.63, *p* = .054, *d* = -0.34, *BF* = 0.701 | 3.56 (1.26) | 3.14 (1.18) |
| 3.1. The tandem breastfeeding mothers achieve a higher score in the authoritative parenting style questionnaire than the non-tandem breastfeeding mothers. | *t*(88) = 0.56, *p* = .711, *d* = 0.12, *BF* = 0.254 | 5.2 (0.4) | 5.24 (0.33) |
| 3.2. The tandem breastfeeding mothers achieve a lower score in the authoritarian parenting style questionnaire than the non-tandem breastfeeding mothers. | *t*(88) = 1.73, *p* = .044, *d* = 0.36, *BF* = 0.816 | 1.79 (0.56) | 1.98 (0.49) |
| 4.1. The tandem breastfeeding mothers achieve a higher score in an attachment parenting style questionnaire than the non-tandem breastfeeding mothers. | *t*(88) = -3.28, *p* = .001, *d* = -0.69, *BF* = 21.118 | 0.78 (0.04) | 0.75 (0.05) |
| 5.1. Tandem breastfeeding mothers experience breastfeeding the older child after the birth of the younger child as more unpleasant than breastfeeding the younger child. | *t*(47) = 7.25, *p* < .001, *d* = 1.05 | 4.42 (0.83)  older child after birth sibling  5.33 (0.57)  younger child |  |
| 5.2. Tandem breastfeeding mothers experience breastfeeding their older child before the birth of the younger child as more pleasant than after the birth. | *t*(47) = 3.36, *p* = .001, *d* = 0.48 | 4.89 (0.85)  older child before birth sibling  4.42 (0.83)  older child after birth sibling |  |
| 5.3. Tandem breastfeeding mothers experience breastfeeding their older child as more pleasant (before the birth of the second child) than the non-tandem breastfeeding mothers. | *t*(88) = 0.42, *p* = .662, *d* = 0.09, *BF* = 0.239 | 4.89 (0.85) | 4.96 (0.76) |
| 5.4. Non-tandem breastfeeding mothers would rather return to breastfeeding another child in the same way as they breastfeed/have breastfed their current children than tandem breastfeeding mothers. | *t*(58.10) = -2.44, *p* = .991, *d* = -0.53, *BF* = 3.568 | 5.4 (0.98) | 4.57 (1.99) |
| 6.1. Tandem breastfeeding mothers experience breastfeeding two children as more energy-consuming than breastfeeding one child. | *t*(47) = -3.31, *p* = .001, *d* = -0.48 | 2.29 (0.88)  before  2.71 (1.13)  after |  |
| 6.2. Tandem breastfeeding mothers experience breastfeeding after the birth of the younger child as more energy-consuming than non-tandem breastfeeding mothers. | *t*(88) = 0.47, *p* = .680, *d* = 0.10, *BF* = 0.244 | 2.71 (1.13) | 2.82 (1.02) |
| 6.3. Tandem breastfeeding mothers have a stronger feeling of sacrificing themselves for their child than non-tandem breastfeeding mothers. | *t*(88) = -0.82, *p* = .207, *d* = -0.17, *BF* = 0.297 | 2.81 (1.51) | 2.55 (1.55) |
| 7.1. Tandem breastfeeding mothers experience breastfeeding as more practical than non-tandem breastfeeding mothers. | *t*(61.84) = -2.27, *p* = .013, *d* = -0.49, *BF* = 2.411 | 5.72 (0.44) | 5.4 (0.8) |
| 8.1. Non-tandem breastfeeding mothers less tired after the birth of the younger child than Tandem breastfeeding mothers. | *t*(88) = -1.78, *p* = .960, *d* = -0.37, *BF* = 0.874 | 3.29 (1.54) | 2.76 (1.25) |
| 8.2. Non-tandem breastfeeding mothers sleep more hours per day after the birth of the younger child than Tandem breastfeeding mothers. | *t*(88) = -1.85, *p* = .966, *d* = -0.39, *BF* = 0.981 | 6.04 (1.66) | 5.49 (1.07) |
| 9.1. Tandem breastfeeding mothers feel less guilty about weaning than non-tandem breastfeeding mothers. | *t*(59) = -0.92, *p* = .820, *d* = -0.24, *BF* = 0.394 | 4.69 (1.05) | 4.34 (1.52) |
| 9.2. Tandem breastfeeding mothers experience weaning as more stressful than non-tandem breastfeeding mothers. | *t*(59) = -1.42, *p* = .081, *d* = -0.36, *BF* = 0.633 | 2.89 (1.63) | 2.24 (1.69) |
| 10.1. Tandem breastfeeding mothers are more likely to experience nausea during pregnancy than non-tandem breastfeeding mothers. | *t*(88) = 0.14, *p* = .555, *d* = 0.03, *BF* = 0.223 | 3.16 (1.02) | 3.18 (1.14) |
| 10.2. Tandem breastfeeding mothers experience more feelings of anger and aggression towards their older child during pregnancy than non-tandem breastfeeding mothers. | *t*(81.48) = -2.60, *p* = .006, *d* = -0.54, *BF* = 3.496 | 2.15 (1.41) | 1.5 (0.92) |
| 10.3. Tandem breastfeeding mothers show more anger during pregnancy than non-tandem breastfeeding mothers. | *t*(88) = -0.68, *p* = .250, *d* = -0.14, *BF* =0.271 | 2.06 (1.21) | 1.88 (1.33) |
| 10.4. Tandem breastfeeding mothers experience the pregnancy as more stressful than non-tandem breastfeeding mothers. | *t*(88) = 0.69, *p* = .755, *d* = 0.15, *BF* = 0.273 | 3 (1.37) | 3.21 (1.57) |
| 11.1. Tandem breastfeeding mothers feel like their breastfeeding behavior is less accepted by their environment than non-tandem breastfeeding mothers. | *t*(88) = 5.36, *p* < .001, *d* = 1.13, *BF* = 20297.12 | 3.54 (0.76) | 4.31 (0.59) |
| 12.1. Children who were tandem breastfed show greater cooperative skills than children who were not tandem breastfed. | *t*(88) = 0.56, *p* = .713, *d* = 0.12, *BF* = 0.255 | 4.34 (0.57) | 4.41 (0.59) |
| 12.2. Children who were tandem breastfed can share better than children who were not tandem breastfed. | *t*(88) = -0.45, *p* = .326, *d* = -0.10, *BF* = 0.242 | 4.52 (0.95) | 4.43 (0.92) |
| 13.1. The partners of non-tandem breastfeeding mothers want to be more involved in the care of the child than the partners of tandem breastfeeding mothers. | t(84) = 0.53, *p* = .300, *d* = 0.11, *BF* = 0.255 | 4.55 (1.3) | 4.69 (1.12) |
| 13.2. Tandem breastfeeding mothers feel more supported by their partners in breastfeeding than non-tandem breastfeeding mothers. | *t*(84) = 0.17, *p* = .567, *d* = 0.04, *BF* = 0.229 | 5.56 (0.85) | 5.59 (0.75) |
| 14.1. Mothers who breastfeed in tandem feel less well advised about breastfeeding than mothers who have not breastfed in tandem. | *t*(88) = 2.51, *p* = .007, *d* = 0.53, *BF* = 3.377 | 3.53 (0.75) | 3.99 (1.01) |
| 15.1. Children who are not breastfed in tandem prefer to eat more complementary food than children who are breastfed in tandem. | *t*(88) = 1.33, *p* = .093, *d* = 0.28, *BF* = 0.483 | 3.67 (1.33) | 4.05 (1.38) |
| 15.2. Children who are not breastfed in tandem prefer to eat more normal food than children who are breastfed in tandem. | *t*(88) = 1.43, *p* = .078, *d* = 0.30, *BF* = 0.542 | 4.37 (0.91) | 4.62 (0.73) |
| 16.1. Tandem breastfeeding mothers have a higher socioeconomic status than non-tandem breastfeeding mothers. | *t*(86) = 0.92, *p* = .821, *d* = 0.20 , *BF* = 0.325 | 2078.41 (755.47) | 2226.76 (747.94) |
| 16.2. There is a positive correlation between the socioeconomic status and the duration of breastfeeding. | *r* = -.22, *p* = .958, *BF* = 0.510 (older child);  *r* = -.46, *p* = .993, *BF* = 1.681 (younger child) |  |  |
| 1. There is a relationship between the country of origin and tandem nursing (Exploratory analysis) | *chi*^2^(3) = 9.84, *p* =.020 |  |  |
| 2. There is a relationship between the highest level of education and tandem-nursing (Exploratory analysis) | *chi^2^* (7) = 9.91, *p* = .194 (mother);  *chi^2^*(8) = 5.33, *p* = .722 (partner) |  |  |

*Note*. Results according to APA specifications
